# Role of Hedgehog Signaling in Vasculature Development, Differentiation, and Maintenance

**DOI:** 10.3390/ijms20123076

**Published:** 2019-06-24

**Authors:** Candice Chapouly, Sarah Guimbal, Pierre-Louis Hollier, Marie-Ange Renault

**Affiliations:** Biology of Cardiovascular Diseases, Universite Bordeaux, U1034, CHU de Bordeaux, F-33604 Pessac, France; candice.chapouly@inserm.fr (C.C.); sarah.guimbal@gmail.com (S.G.); p-l_hollier@hotmail.fr (P.-L.H.)

**Keywords:** Hedgehog, vasculogenesis, angiogenesis, endothelium, blood–brain barrier

## Abstract

The role of Hedgehog (Hh) signaling in vascular biology has first been highlighted in embryos by Pepicelli et al. in 1998 and Rowitch et al. in 1999. Since then, the proangiogenic role of the Hh ligands has been confirmed in adults, especially under pathologic conditions. More recently, the Hh signaling has been proposed to improve vascular integrity especially at the blood–brain barrier (BBB). However, molecular and cellular mechanisms underlying the role of the Hh signaling in vascular biology remain poorly understood and conflicting results have been reported. As a matter of fact, in several settings, it is currently not clear whether Hh ligands promote vessel integrity and quiescence or destabilize vessels to promote angiogenesis. The present review relates the current knowledge regarding the role of the Hh signaling in vasculature development, maturation and maintenance, discusses the underlying proposed mechanisms and highlights controversial data which may serve as a guideline for future research. Most importantly, fully understanding such mechanisms is critical for the development of safe and efficient therapies to target the Hh signaling in both cancer and cardiovascular/cerebrovascular diseases.

## 1. Introduction

The Hedgehog (Hh) family of morphogens, which includes Sonic Hedgehog (Shh), Indian hedgehog (Ihh), and Desert Hedgehog (Dhh), was identified nearly four decades ago in Drosophila as critical regulators of cell fate determination during embryogenesis [1].

Shh is the most widely expressed and studied. It has been implicated in the development of multiple organs including the central nervous system (CNS), lungs, foregut, heart, and limbs [2] by participating in axis orientation and orchestrating branching morphogenesis. The role of Ihh and Dhh is restricted to a limited number of organs. While Ihh participates in the development of the gut, bones, and kidneys [3,4], Dhh orchestrates the development of testis and peripheral nerves [5,6]. In adults, the Hh signaling is generally silent, but participates in tissue maintenance and regeneration by controlling stem cell renewal and differentiation in the brain subventricular zone and in hair follicles [7]. Besides, inappropriate activation of Hh signaling has been linked to several disparate human cancers including basal cell carcinoma, medulloblastoma, lung cancer, and pancreatic adenocarcinoma [8].

The role of the Hh signaling in vascular biology has first been highlighted in embryos by Pepicelli et al. in 1998 [9] and Rowitch et al. in 1999 [10]. Indeed, while the first study reveals a sparser lung vascular network in Shh deficient embryo, the second study shows that ectopic Shh expression induces hypervascularization suggesting a proangiogenic role of the Hh signaling. Since then, the proangiogenic role of Hh ligands has been confirmed in adults especially in pathologic conditions [11,12,13]. Moreover, Hh ligands have been shown to regulate blood vessel maturation [14], integrity [15], and arterial differentiation [16]. As a consequence, the therapeutic potential of Hh signaling agonists for vascular diseases is of growing interest [17,18,19,20,21,22]. However, molecular and cellular mechanisms underlying the role of the Hh signaling in vascular biology remain poorly understood. The present review summarizes the current knowledge and discrepancies regarding the role of Hh signaling in vasculature development, differentiation, and maintenance, which is important to consider for future research directions and therapeutic perspectives.

## 2. Hedgehog Signaling and Regulation

### 2.1. Regulation of Hh Ligand Secretion

**Figure 1:**
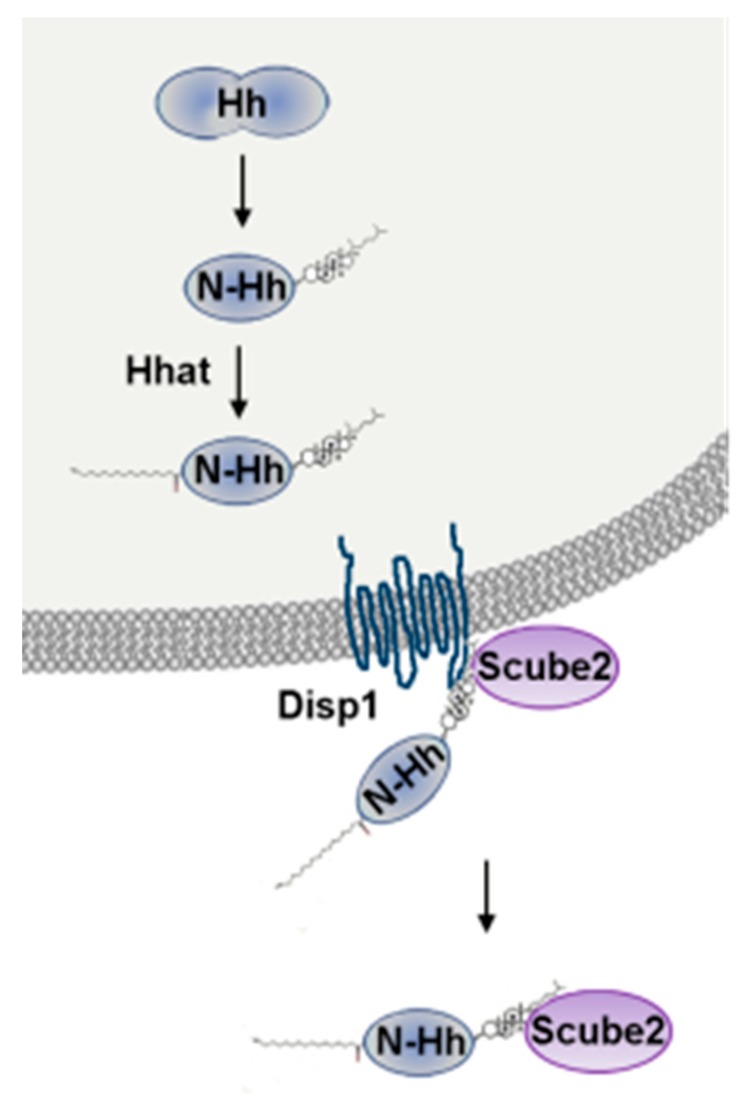
Shh post-transcriptional modification and secretion. Shh is synthetized as a full-length, 45 kDa protein. An autocatalytic reaction removes the carboxy-terminal domain and attaches a cholesterol moiety to the newly exposed carboxy-terminus. Then, Hhat catalyzes the addition of a palmitate to the amino-terminus [23]. Secretion and solubility of Shh depends on Disp1 and Scube2.

Shh is synthetized as a preprotein of which the signal sequence is first cleaved to produce a full-length unmodified form. An autocatalytic reaction removes then the carboxy-terminal domain and attaches a cholesterol moiety to the newly exposed carboxy-terminus. Shh is further modified by Hedgehog acyltransferase (Hhat), which catalyzes the addition of a palmitate to the amino-terminus [23] (Figure 1). Ihh and Dhh processing have been poorly investigated, and may differ. As a matter of fact, Dhh is suggested not to undergo efficient autocatalytic cleavage [24].

Secretion and solubility of cholesterol-modified Hh ligands depend on the transmembrane protein Disp1 (dispatched RND transporter family member 1) and the cell surface protein Scube2 (signal peptide, CUB domain and EGF-like domain containing 2) (Figure 1) [25]. Both Disp1 and Scube2 bind the cholesterol-anchor of Shh.

### 2.2. Hh Signaling

**Figure 2:**
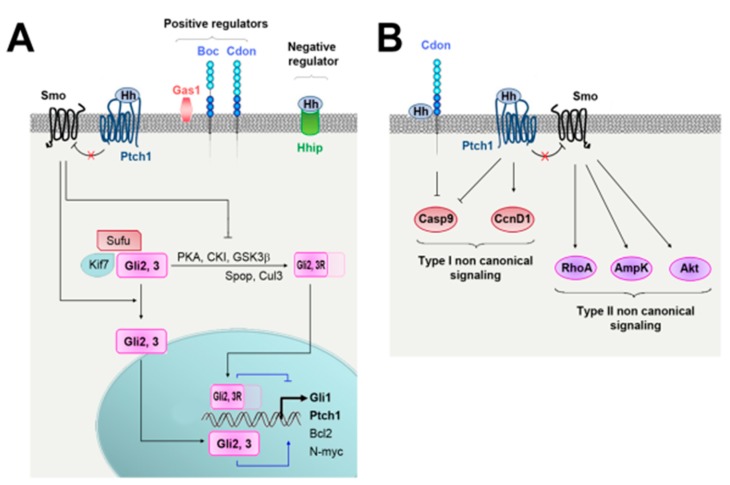
(**A**) Hh canonical signaling. In the absence of Hh ligands, Smo is inhibited by Ptch1 and Gli transcription factors are associated with SUFU negative regulator of hedgehog signaling (Sufu) and kinesin family member 7 (Kif7). This last complex promotes Gli3 and Gli2 phosphorylation by cAMP dependent protein kinase (PKA), casein kinase 1 (CK1), and glycogen synthase kinase 3 beta (GSK3. Once phosphorylated, Gli2 and Gli3 are processed by speckle type BTB/POZ protein (Spop)/cullin 3 (Cul3) ubiquitin ligase complex to generate Gli2R and Gli3R (repressor forms) respectively. Hh ligands binding to Ptch1 leads to Smo activation, which prevents Gli2 and Gli3 cleavage. Full-length Gli2 and Gli3 may then translocate to the nucleus and activates transcription. (**B**) Hh noncanonical signaling. Hh binding to Ptch1 or Cdon may, independently on Smo, promote cell survival or proliferation by modulating Caspase 9 (Casp9) or Cyclin D1 (CcnD1) activity, respectively. This is what is called type I noncanonical signaling. Alternatively, Hh ligands may activate PI3K/Akt, RhoA/ROCK or AMPK, via Smo, but independently on Gli transcription factors. This is what is called type II noncanonical signaling.

The interaction of the Hh proteins with their specific receptor Patched-1 (Ptch1) de-represses the transmembrane protein Smoothened (Smo), which activates downstream pathways, including the Hh canonical pathway leading to the activation of Gli transcription factors and so-called Hh noncanonical pathways, which are independent of Smo and/or Gli (Figure 2) [26].

Activation of the Hh canonical pathway promotes cell survival and proliferation through the regulation of Bcl2, N-myc and CyclinD1 while the noncanonical signaling has been involved in cytoskeleton changes and cell migration [26]. Hh-induced paracrine signaling on adjacent cells is the most common mode of pathway transduction, although Hh has also been proposed to signal in an autocrine manner.

### 2.3. Regulation of Hh Signaling

Hh binding to Ptch1 is regulated by several coreceptors. Among these, Cell adhesion molecule-related/downregulated by oncogenes (Cdon), Brother of Cdon (Boc) and Growth arrest specific 1 (Gas1) are suggested to promote Hh ligand interaction with Ptch1 while Hedgehog interacting protein (Hhip) inhibits it [27] (Figure 2A).

Beside, Hh signaling activity has been shown to depend on the primary cilium. Indeed, several Hh signaling pathways elements including Smo, Sufu, Kif7, Gli2, and Gli3 have been located at the primary cilium and disruption of genes encoding for cilia proteins such as intraflagellar transport proteins (Ift), talpid3, and *Dzip1/Iguana* recapitulates most features of Shh deficiency [28].

## 3. Vascular Development

### 3.1. Yolk Sac Vascularization

**Figure 3:**
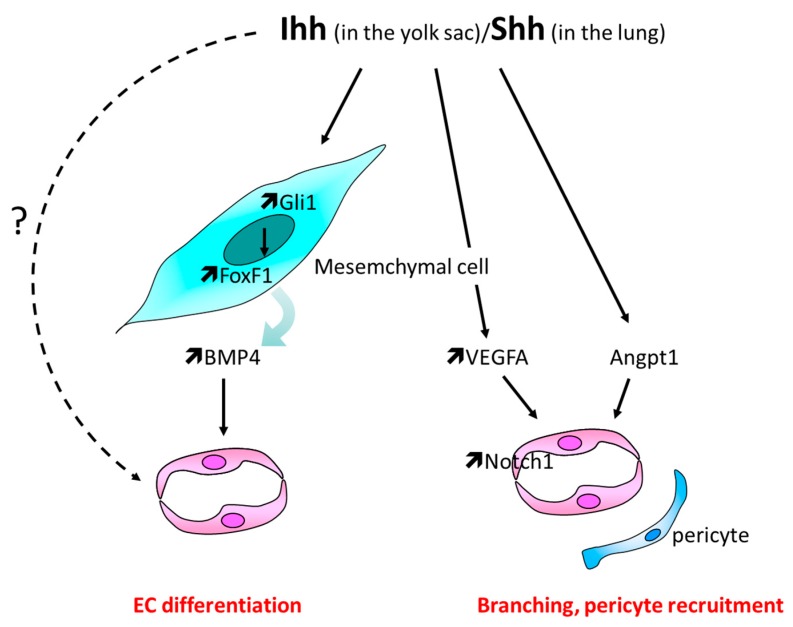
Schema representing the main cellular events involved in Hh-induced vasculogenesis and primary vascular plexus remodeling. Hh ligands promote EC differentiation indirectly via BMP4 upregulation in mesenchymal cells, while vascular remodeling, i.e., branching and pericyte recruitment, depends on Vegfa and/or Angpt1.

Hh signaling has been shown to orchestrate angiogenesis in the yolk sac as Smo^KO^ embryos fail to form yolk sac blood vessels [29]. Ihh, secreted from the primitive endoderm, seems to be the main ligand responsible for this effect [30]. Nevertheless, while Smo^KO^ embryos do not form any blood vessels, Ihh^KO^ embryos do form ones which fail to undergo vascular remodeling, i.e., ramification into large and small branches and pericyte coverage [29,31]. This suggests that either Shh and/or Dhh also participate in yolk sac vascularization or that Ihh effects are partly compensated by Shh and/or Dhh in the absence of Ihh. Hh signaling has been shown to promote vasculogenesis through forkhead box F1 (FoxF1) and bone morphogenetic protein 4 (BMP4) [30,32], while vessel remodeling seems to depend on (vascular endothelial growth factor) VEGF, kinase insert domain receptor (KDR/Flk-1), and notch receptor 1 (Notch-1) [31] (Figure 3).

At the cellular level, it is still not clear which cell types respond to Ihh signals: in vitro, endothelial cells (EC) themselves are suggested to respond to Ihh since C166 cells, a mouse yolk sac EC line, respond to Shh recombinant protein by overexpressing Gli1 and Ptch1 and migrating more. In these cells, Shh also increases expression of neuropilin 1 (Nrp1), Kruppel like factor 4 (Klf4), jagged canonical Notch ligand 1 (Jag1), and collagen type IV alpha 1 chain (Col4a1), major factors implicated in EC biology [33].

### 3.2. Lungs Vasculature Development

Shh-deficient mouse lungs have first been reported as poorly vascularized by Pepicelli et al. in 1998 [9]. Later on, altered vasculature characterized by a sparse network with large gaps between capillaries has been outlined in both Shh^KO^ and Smo^KO^ mouse embryos especially in the distal part of the lungs [34,35]. Notably, Vegfa expression depends on Smo in the distal part of the lung while its expression in the subepithelial mesenchyme appears to be less dependent on the Hh signaling [35]. However, according to Van Tuyl et al., the pulmonary vascular bed is decreased in Shh^KO^ embryos, but appropriate to the decrease in airway branching. In the same study, Vegfa expression is reportedly not different from that of control lung [36] and early vascular development in lungs, mediated by Vegf/Kdr signaling is then suggested to proceed normally in Shh^KO^ embryos likely because of possible compensatory effects from the other Hh ligands. On the contrary, vascular stabilization is defective because of angiopoietin 1 (Angpt1) downregulation [36].

At the cellular level, the lung capillary network development does not depend on a direct effect of Shh on EC since it is normal in Smo^ECKO^ lungs [35]. In contrast, it depends on Shh-induced FoxF1 expression, via Gli-binding sites in unidentified cells, most likely of mesenchymal type [37] (Figure 3).

### 3.3. Formation of the Aorta and Intersomitic Vessels

**Figure 4:**
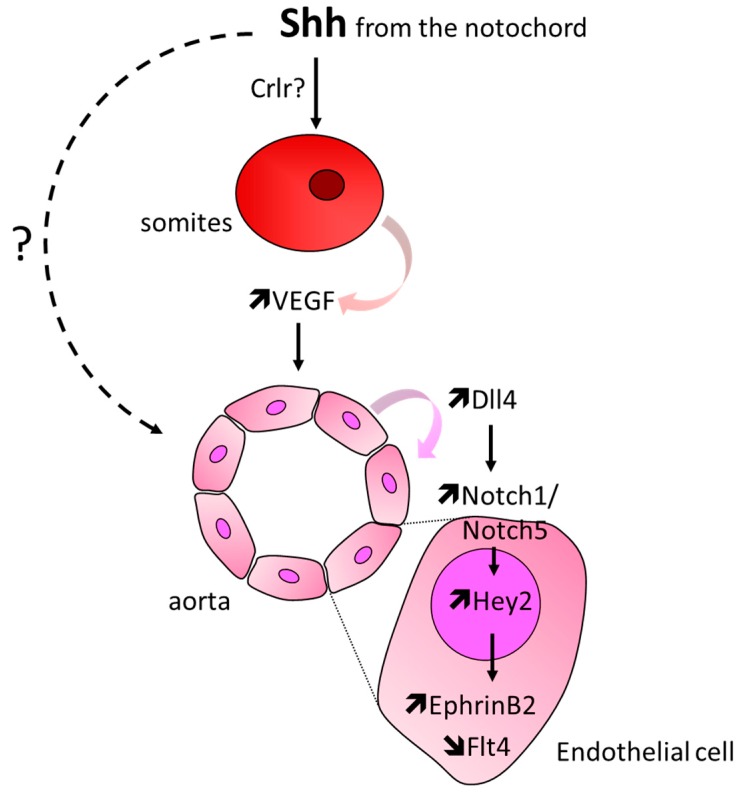
Schema representing the main cellular events involved in Hh-induced arterial differentiation. Briefly, Shh produced by the notochord upregulates Vegfa in somites, which, in turn, increases Notch signaling in ECs, and subsequently promotes the expression of the arterial marker EphrinB2.

Hh signaling is also necessary for the formation of the aorta. In avian embryos, Smo and Shh inhibition using cyclopamine and 5E1 blocking antibodies, respectively, are reported to impair both aorta formation and remodeling [38,39,40] and activation of the Hh pathway with SAG leads to the formation of an enlarged aorta. It has also been highlighted that angioblasts of Smo^KO^ mouse embryos fail to organize and form the aorta especially in the anterior two-thirds of the embryo [38] while over activation of the Hh pathway through deletion of Ptch1–a negative regulator of Hh signaling results in a dilated dorsal aorta [41]. Consistently, studies performed in zebrafish embryos report that Sonic-you (Syu) (Shh ortholog), You-too (yot) (Gli2 ortholog), Smo mutant, or cyclopamine-treated zebrafishes fail to form the dorsal aorta [16,42,43] and that administration of 5E1 Hh-blocking antibodies result in many vascular defects including delayed fusion of the dorsal aorta and hemorrhage [39]. Besides, Shh promotes arterial differentiation, since Syu, yot, and Smo mutant zebrafishes fail to express EphrinB2, an arterial-specific marker [16] (Figure 4).

Shh is proposed to promote tube formation and arterial identity via Vegfa [16,41,44]; more specifically, Shh may induce Vegfa expression in somites via calcitonin receptor-like receptor (crlr) [45] which in turn promotes Notch5 expression in ECs. Activation of Noth signaling, subsequently, inhibits Flt4 and promotes EphrinB2 expression [16]. As an alternative, Shh is suggested to be necessary for angioblasts to organize and to promote arterial identity by repressing venous cell fate in angioblasts [43]. This is supported by the fact that angioblasts and later on, aortic ECs express Ptch1, Ptch2, and Smo [38]. 

Artery and vein identity was reported to be established normally in *igu^fo10a^* mutants (The zebrafish genetic mutant iguana (igu) has defects in the ciliary basal body protein Dzip1, causing improper cilia formation and disruption of Hh signaling [46]. 

### 3.4. Involvement of Hh Signaling in the Setting of Other Vascular Bed Development

Hh signaling is involved in the vascular development of other organs notably the retina, brain, skeleton, and heart.

The retina: Hh signaling is implicated in retinal angiogenesis, as cyclopamine inhibits retinal angiogenesis [47], and is shown to be necessary for both retinal EC and pericyte survival. Indeed, Shh promotes Ptch1, Gli2, Notch1, Notch3, Bcl2, and Bclxl, but inhibits Bax expression in retinal ECs, while it promotes Ptch1, Gli2, Notch1, and Bcl3 in pericytes. However, while pulsatile flow promotes Hh signaling and retinal EC survival, it inhibits it in retinal pericytes [48]. 

The brain: Gli2 deficient embryos have been reported to lack the entire brain basilar artery [49] and Shh, produced by the hindbrain choroid plexus epithelial cells, to promote hind brain vascularization. Shh more likely signals to pericytes but not ECs to, since only pericytes are reported to express Ptch1 in this study [50]. 

The skeleton: Ihh promotes skeletal blood vessel 3D organization and stability [51] and Shh recombinant protein enhances angiogenesis and osteogenesis in a co-culture system consisting of primary osteoblasts and outgrowth ECs [52]. In bones, Ihh is proposed to act downstream of the Vegf signaling since the Vegfa/Vegfr2 signaling stimulates its expression and activity [53].

The heart: Shh is necessary for FGF9-induced Vegfa, Vegfb, Vegfc, and Angpt2 expression in cardiomyocytes and perivascular cells [54] and the Hh signaling to cardiomyocytes is required for the development of coronary veins, while Hh signaling to perivascular cells seems necessary for coronary arterial growth [55]. 

Somite: In avian embryos, inhibition of Smo and Shh compromise intersomitic vessel growth [56], while activation of the Hh pathway with SAG leads to a densified plexus. Moreover, the number of EC filopodia is found to correlate with Hh signaling activity since the number of filopodia decreases in cyclopamine-treated embryos, while increases in the SAG-treated embryos [40]. At a molecular level, growth of intersomitic vessels seems to be dependent on Vegfa, but independent on Notch or BMP [56]. 

Finally, ectopic Shh overexpression in the dorsal neural tube was shown to induce spinal cord hypervascularization [10], while ectopic expression of a constitutively active form of Smo (SmoM2) in the ovaries was shown to increase the density of CD31+ endothelial tubes in newborn mice [57].

### 3.5. Controversial Data

Even though Hh signaling appears to promote vascular development in the yolk sac, lungs, bones, heart, retina, and ovaries, organ specificity exists and contradicting observations have been made: for example, superficial vessel development in the zebrafish eye is increased due to excessive vessel sprouting in Smo deficient fish [58], while, in accordance with previous reports, vegf showed a clear downregulation. It has also been described that Talpid3 (a gene necessary for the Hh signaling that participates in primary cilium formation)-deficient chicken embryos display severe vascular defects including enlarged and more numerous blood vessels. Moreover, electron-dense junctions between talpid3 ECs appeared less well-defined which is associated with hemorrhage and edema [59]. The expression of Vegfa is unchanged, while Vegfd, Rigf (retinoic-acid induced growth factor, a chicken member of Vegf family), and angiopoietin 2a (Angpt2a) are overexpressed. Nrp1 is expressed by both veins and arteries and Nrp2, normally expressed in veins, is absent. Moreover, this study reported that ectopic expression of Shh leads to a decreased capillary density, a transient upregulation of Nrp1 and Angpt2 but no modulation of neuropillin 2 (Nrp2) [59].

## 4. Postnatal Angiogenesis

In addition to its role in embryo vascularization, the Hh signaling has also been identified as regulating postnatal angiogenesis, especially in the setting of ischemia [11] and cancer [60].

### 4.1. In the Setting of Ischemia

#### 4.1.1. Role of Hh-Signaling in Ischemia-Induced Angiogenesis in the Hindlimb

The Hh signaling has been shown to be reactivated in ischemic conditions especially in the hindlimb muscle. Shh [61,62], Gli1 [63,64], Gli2, and Gli3 [65] were shown to be strongly upregulated in the ischemic muscle compared to the contralateral nonischemic muscle. Reactivation of Hh signaling is suggested to promote revascularization of ischemic tissues since systemic administration of Hh-blocking antibodies (5E1) decreases capillary density and reperfusion of the ischemic limb [61]. Besides, overactivation of Hh signaling by ectopic administration of a Shh-expressing vector [66], Shh-carrying microparticles [67], or the Smo-agonist SAG [68] increases capillary density in the ischemic muscle.

Reactivation of Hh signaling is suggested to be impaired in the setting of aging since Gli1 expression in the ischemic muscle is diminished in aged mice [63,64]. Dhh and Smo, but not Shh expression, were shown to be downregulated in aged mice [64]. Because of the proangiogenic properties of Hh ligands, impaired activation of Hh signaling in aged mice is proposed to compromise ischemia-induced angiogenesis. This paradigm is supported by the fact that administration of Hh ligands either systemically [11] or locally [63,64] increases capillary density, promotes ischemic muscle perfusion, and limb salvage in aged mice. 

##### Controversial Data

It still remains unclear which Hh ligand(s) regulate ischemia-induced angiogenesis. Indeed, angiogenesis has been shown to be transiently accelerated in Shh iKO mice [66] suggesting that endogenous Shh has antiangiogenic properties rather than proangiogenic ones. Besides, we found that angiogenesis is impaired in Dhh constitutive KO mice [62] but this is more likely the result of an impaired peripheral nerve development since the same phenotype is recapitulated in denervated mice but not in mice in which Dhh KO is induced in adult mice (unpublished data). Therefore, according to the results obtained so far, neither Shh nor Dhh seem to be promoting angiogenesis in the setting of hindlimb ischemia. Another option is that Hh signaling may be activated through noncanonical signaling, regardless of any Hh ligands. For example, Gli3, which is necessary for ischemia-induced angiogenesis [65], is upregulated by E2F1 in myoblasts [69]. 

**Figure 5:**
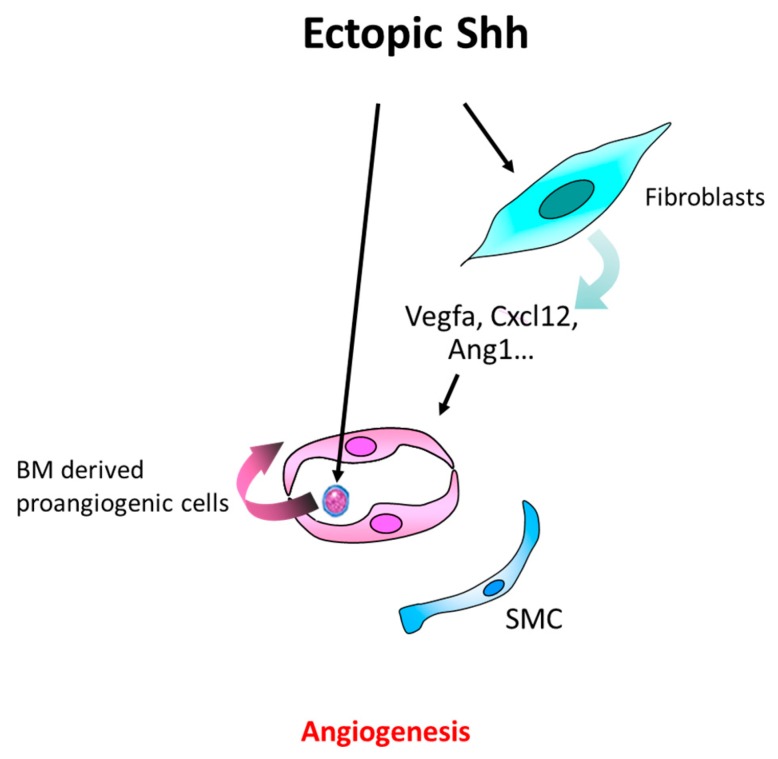
Schema representing the main cellular events underlying the proangiogenic effect of Shh therapy in the setting of ischemia. When administered ectopically in ischemic tissues, Shh promotes angiogenesis indirectly by upregulating Vegfa, Cxcl12, and Angpt1 in fibroblasts and by recruiting bone marrow-derived proangiogenic cells.

The Hh signaling to ECs does not participates in ischemia-induced angiogenesis since angiogenesis occurs normally in Smo^ECKO^ mice [62,70]. On the contrary, Hh ligands are proposed to promote angiogenesis indirectly by increasing proangiogenic factor expression (Vegfa, Angpt1, Angpt2) in fibroblasts [11,63]. Hh ligands were also shown to promote recruitment of bone marrow derived proangiogenic cells in ischemic tissues [63] (Figure 5) and Hh signaling may promote angiogenesis by regulating myogenesis. Indeed, Gli3^ECKO^ does not alter ischemia-induced angiogenesis, while angiogenesis is impaired in mice in which Gli3 expression is disrupted in myoblasts [69]. Moreover, Gli3 regulates Angpt1 and thymidine phosphorylase (TYMP) expression in myoblasts. Finally, Shh has been shown promotes myogenesis in adults in both cardiotoxin and mechanical crush-induced muscle injury models [71,72]. Shh, when administered ectopically, does not recapitulate endogenous Shh effects [66], suggesting that ectopic Hh ligand and endogenous Hh ligand control ischemia-induced angiogenesis through distinct mechanisms, e.g. ectopically administered Shh increases Vegfa expression in fibroblasts, while endogenous Shh decreases C–C motif chemokine ligand 2 (Ccl2) expression in myoblasts which results in a decreased macrophage invasion and diminished macrophage-derived Vegfa levels in the ischemic muscle [66].

#### 4.1.2. Role of Hh Signaling in Other Ischemic Tissues

The proangiogenic properties of the Hh ligands have been confirmed in other ischemic tissues including the heart [73,74], brain [75,76,77], skin [78], and peripheral nerves [79]. In each of these organs, administration of Shh (or a Smo agonist) increases capillary density after an ischemic insult. In line with the results obtained in the limb skeletal muscle, Shh-induced angiogenesis in the heart does not involve activation of Hh signaling in ECs [70]. On the contrary, Shh-induced angiogenesis has been associated with increased Vegfa levels in all these organs [73,76,78]. The role of the Vegfa in Shh-induced angiogenesis has been proven both in the skin [78] and in the brain [76] using anti-Vegfa antibodies and Shh has also been reported to promote the angiogenic capacity of the bone marrow derived cells in the heart [73,80] and in the skin [78].

Once again, the role of the endogenous Hh signaling in the heart is not clear, while Lavine KJ et al. reported that Hh-blocking antibodies administration decreases capillary density and cardiomyocyte survival in the setting of myocardial infarction [81], it has also been shown that cyclopamine administration ameliorates heart function [82].

### 4.2. In the Setting of Cancer

Hyperactivation of the Hh signaling observed in tumors is suggested to promote tumor angiogenesis. In detail, the Hh signaling blockade with Smo antagonists, including GDC-0449 and Cyclopamine, reduces the vascular density of Hh-producing colon cancer xenografts [60] and oral squamous cell carcinoma [83] or melanoma [84]. Conversely, ectopic expression of Shh in low-Hh-expressing DLD-1 xenografts increases tumor vascular density and augments angiogenesis [60] and the tumors implanted in Hhip+/− mice exhibit increased tumor angiogenesis [85]. Finally, high Gli1 expression levels have been correlated with increased microvascular density in Glioma [86] and high KDR expression in triple-negative breast cancers [87] while Ihh expression has been associated with Vegf expression and CD34 staining in hepatocellular carcinoma [88].

**Figure 6:**
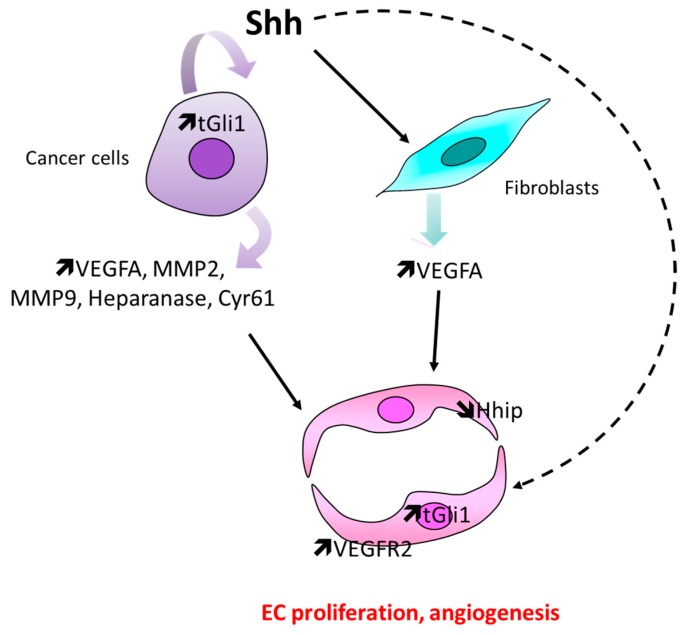
Schema representing the main cellular events involved in Hh-induced tumor angiogenesis. In tumors, Shh, which is mainly produced by cancer cells, may promote angiogenesis either by increasing proangiogenic factor expression in cancer cells themselves, by promoting Vegfa expression in stromal fibroblast, or by promoting EC proliferation directly through Gli1 upregulation.

Hyperactivation of Hh signaling in cancer cells themselves (i.e., Gli1 or Shh overexpression) has been shown to increase proangiogenic factor expression including vegfa [86,89,90], matrix metallopeptidase 2 (MMP2), matrix metallopeptidase 9 (MMP9) [86], and heparanase [91] in glioma cells or cysteine-rich angiogenic inducer 61 (Cyr61) in breast cancer cells [92]. In particular, a novel alternatively spliced, truncated form of GLI1 (but not full-length GLI1) binds Vegfa promoter. Shh produced by cancer cells is proposed as an alternative to promote Vegfa expression in stromal fibroblasts [60,85], which subsequently induces EC proliferation. Moreover, Hh ligands produced by cancer cells are also proposed to modulate EC function directly: Shh is highly expressed in human tongue oral squamous cell carcinoma (OSCC) whereas Ptch1, Gli1 and Gli2 proteins are expressed in the microvascular cells in the tumor invasive front [83,93]. In cultured HUVEC, Shh is reported to promote cell proliferation [84], while tGli1 promotes Vegfr2 expression [87] and Hhip, which is highly expressed in ECs, and is downregulated in ECs undergoing angiogenesis. These results suggest that a reduced expression of Hhip in tumor neovasculature may contribute to an increase Hh signaling within the tumor and may possibly promote angiogenesis [94]. Finally, Shh-derived from adeno-pancreatic cancer cells may promote the angiogenic properties of bone marrow derived progenitor cells [95] (Figure 6).

Apart from being expressed by cancer cells, Hh ligands are also expressed by ECs (Shh and Ihh) [93,96,97], especially in oral squamous cell carcinomas and gliomas, macrophages (Ihh) [93], and astrocytes [96]. Notably, inhibition of endothelial Scube2 suppresses tumor angiogenesis [98] and Shh may be carried by microvesicles especially from oral squamous cell carcinoma [99].

#### Controversial Data

While most studies agree in reporting that hyperactivated Hh signaling in tumors may promote tumor angiogenesis, a few studies have shown opposite results: first, Smo inhibition using IPI-926 has been reported to increase tumor vessel density in pancreatic ductal adenocarcinoma [100]. Shh-deficient tumors have been recently identified as more aggressive and exhibiting undifferentiated histology, increased vascularity, and heightened proliferation features that are fully recapitulated in control mice treated with IPI-926. Furthermore, administration of a Vegfr-blocking antibody selectively improves survival of Shh-deficient tumors, indicating that Hh-driven stroma suppresses tumor growth in part by restraining tumor angiogenesis [101]. Another Smo inhibitor—NVP-LDE225 (erismodegib)—has been shown to restore vascular density in pancreatic ductal adenocarcinoma, to decrease pericyte coverage and to enhance vessel permeability, suggesting an increased proportion of immature microvessels [102].

### 4.3. Other Pathological Angiogenesis

Hh signaling has been involved in atherosclerosis plaque angiogenesis. Insulin resistance adipocyte-derived exosomes (IRADEs) carry Shh, which promotes plaque vulnerability partially by inducing vasavasorum angiogenesis. This is associated with increased Gli1 and Vegfa expression levels [103]. In addition, Shh and Ptch1 are overexpressed in the eye both in retinopathy of prematurity (ROP) [47] and in laser-induced choroidal neovascularization [47,104], promoting pathological angiogenesis; inhibition of the Hh pathway (Cyclopamine) results in reduced angiogenesis and decreased Vegfa and Ptch1 levels, placing Shh activation upstream of Vegfa in experimental retinal angiogenesis [47]. Moreover, in mice with chronic liver injury or mice that underwent acute partial hepatectomy, administration of a Smo antagonist (GDC-0449 or Cyclopamine) prevents liver sinusoidal EC capillarization [105], and a study suggests that Annexin a2 may promote EC proliferation and angiogenesis by increasing Ihh and Gli1 in the setting of rheumatoid arthritis [106].

## 5. Maintenance of Blood Vessel Integrity and Quiescence

### 5.1. At the Blood–Brain Barrier

**Figure 7:**
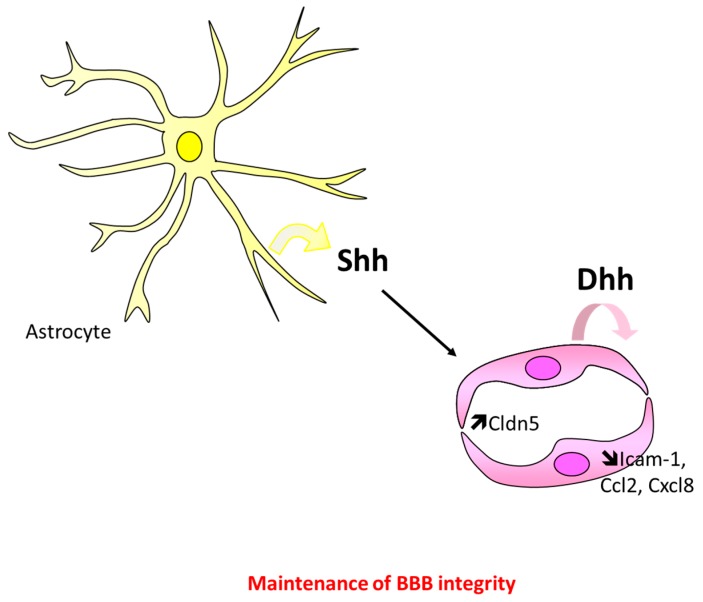
Schema representing the main cellular events underlying Hh maintenance of BBB integrity. Activation of Hh signaling in brain ECs promotes thigh junction integrity by increasing Cldn5 expression and BBB immune quiescence by downregulating Icam1, Ccl2, and Cxcl8. Brain ECs may either respond to Dhh, which is produced by EC themselves in physiological conditions, or to Shh, which is produced by astrocytes in certain pathological conditions.

The critical role of Hh signaling in maintaining BBB integrity has first been highlighted in 2011 by Prat’s laboratory [15]. This study revealed that the Hh signaling promotes BBB integrity both in embryos and in adults, since both Shh^KO^ embryos and cyclopamine-administered adult mice display brain vascular leakage [15]. Interestingly, in this article, the same phenotype is recapitulated in Smo^ECKO^ mice (Tie2-Cre; Smo^Flox/Flox^) demonstrating that ECs are the cells mediating Hh regulation of BBB integrity. Activation of the Hh signaling in ECs decreases BBB permeability and increases trans-endothelial electrical resistance of brain ECs by promoting expression of both tight (Claudin-3, Claudin-5 (Cldn5), Occludin, F11 receptor (Jam-A), (tight junction protein 1) ZO-1) [15,107], and adherens (Cadherin-5 (Cdh5), p120) [15] junction proteins. It is important to note that the upregulation of junction proteins has been associated with increased Gli1, SRY (sex-determining region Y)-box 18 (Sox18) [15,108], and Netrin1 [109] expression. Finally, Hh signaling is suggested to prevent EC activation, since Shh and purmorphamine have been shown to downregulate Ccl2, C-X-C motif chemokine ligand 8 (Cxcl8), and intercellular adhesion molecule 1 (Icam-1) expression in cultured brain ECs resulting in decreased CD4+ T cell adhesion and transmigration [15] (Figure 7).

We recently demonstrated that BBB integrity in adult mice depends on Dhh, which is produced by ECs themselves, using Dhh^ECKO^ mice [110] (Figure 7), and BBB integrity is suggested to depend on Shh whom expression is reportedly modulated in several pathological conditions. Even though the role of astrocyte-derived Shh in maintaining BBB integrity in adults needs to be demonstrated using conditional KO mice, so far, it could be hypothesized that Dhh regulates BBB integrity in physiological conditions while Shh regulates it in certain pathological conditions. Indeed, Dhh is downregulated by inflammatory cytokines [110] while Shh is overexpressed in activated astrocytes in the setting of neuroinflammation (multiple sclerosis) [15], stroke [77,111], and subarachnoid hemorrhage. On the contrary, both Shh and Gli1 are decreased in HIV-associated dementia [112,113] and in the setting of forebrain stab injury [114]. Either ways, administration of Hh signaling agonists (rec NShh, SAG or Purmorphamine) have been shown to increase tight junction protein expression and to decrease BBB permeability [77,111,112,113], while administration of a Smo antagonist increases BBB permeability or brain inflammation [15,115].

These data are supported by few other in vitro studies in which astrocytes were co-cultured with brain ECs. The first study reports that the Smo agonist Purmorphamine decreases *Mycobacterium tuberculosis*-induced BBB disruption. *Mycobacterium tuberculosis* did not affect Shh astrocytic expression, but it decreases Scube2 expression and prevents Shh secretion [108]. In the second study, the Wip1 phosphatase prevents BBB breakdown and production of proinflammatory cytokines by increasing Shh and Gli1 expression [116]. Another article highlights the capacity of oxLDL to promote brain EC apoptosis by decreasing Shh-induced autocrine signaling [117]. As an alternative, Shh is proposed to promote BBB integrity by increasing Angpt1 expression autocrinally in astrocytes [111].

#### Controversial Data

A recent paper studying glioblastoma highlighted an opposite effect of the Hh signaling at the BBB. This study reports that “patient-derived glioblastoma-initiating cells” secrete Dhh, which interacts with endothelial Ptch2 via a paracrine mechanism to exacerbate BBB permeability [118]. Moreover, ectopic Shh overexpression in the dorsal neural tube has been shown to induce hypervascularization and hemorrhage in the spinal cord [10]. Another contradictory study reports that Shh carried by micro particles upregulated Icam-1 [119].

### 5.2. At the Blood Nerve Barrier

Activation of the Hh signaling in ECs is also shown to promote blood nerve barrier (BNB) integrity since Smo^ECKO^ mice display abnormal endoneurial capillary permeability and nerve inflammation [120]. Schwann cell-derived Dhh is suggested to be responsible for this effect [120,121]. As for the data obtained in the CNS, the Hh signaling is suggested to promote BNB integrity by increasing Cldn5 and Ocln expression and by decreasing Ccl2 and interleukin 1 beta (Il1-β) expression [120,122].

Moreover, the increased BNB permeability associated with diabetic neuropathy has been shown to result from Dhh downregulation [120]. In the setting of chronic constriction injury (CCI), Shh expression is increased transiently, whereas Gli1 and Ptch1 expression are both decreased [122,123]. Finally, cyclopamine local administration mimic chronic constriction injury-induced vascular alterations including nerve inflammation and BNB opening [122] confirming the essential role of Hh signaling in promoting BNB integrity and preventing nerve inflammation.

### 5.3. Outside of the Nervous System

We recently reported that blood vessel integrity depends on Hh signaling not only within the central and peripheral nervous system, but also in other organs including the heart and the lung. Endothelial adherens junction integrity and immune quiescence depends on Dhh expression by ECs themselves. Indeed, Dhh^ECKO^ mice display spontaneous vascular leakage and exacerbated LPS-induced neutrophil recruitment in the lung. Notably, Dhh is a downstream target of Klf2 which promotes interaction of the Cdh5 with its partner the β-catenin and decreases Icam-1 and vascular cell adhesion molecule 1 (Vcam-1) expression; nevertheless Dhh effects are more likely independent on Gli transcription factors since neither Gli1 nor Gli2 expression is modulated in Dhh KO ECs [110]. Interesting data show that Dhh, Shh, Gli1 and Ptch1 expression in ECs are downregulated by proinflammatory signals including tumor necrosis factor (TNFα) and lipopolysaccharide (LPS), which contribute to LPS-induced EC dysfunction since treatment with Dhh or SAG can prevent TNFα-induced increased capillary permeability and Vcam-1 expression [110,124].

Hh regulation of blood vessel survival, especially in the heart, is also proposed to depend on the Hh signaling activity in cardiomyocytes and on the resulting overexpression of Vegfa, Vegfb, Vegfc, Angpt1, and Angpt2 [81].

Finally, in the retina of Akika diabetic mice, the deceased expression of Shh has been associated with decreased Angpt1 expression, increased capillary permeability, and pericyte loss [125].

Apart from regulating endothelial intercellular junctions and endothelial immune quiescence, Hh ligands may also regulate vasoactive properties of ECs since administration of microparticles carrying Shh have been shown to promote both nitric oxide synthase 3 (NOS3) expression and NOS3 phosphorylation [67,126]. Moreover, microparticles carrying Shh correct ischemia/reperfusion [126] or Angiotensin-II induced-impaired vasorelaxation [127] and increased Hhip expression at the surface of endothelial-derived microparticles in the setting of acute graft-versus-host disease (aGVHD) promotes EC apoptosis, decreases NOS3 expression, and increases expression of ICAM-1 and VCAM-1 [128].

### 5.4. Molecular Mechanism Involved in Hh-Induced Maintenance of Endothelium Integrity

While Hh regulation of angiogenesis is mediated via Hh-induced upregulation of Vegfa in fibroblast-like cells, Hh regulation of blood vessel integrity seems to be cell-autonomous. Indeed, both Smo^ECKO^ and Dhh^ECKO^ mice are reported to display abnormal vessel permeability and several studies report expression of Hh ligands by ECs themselves: for instance, Shh is expressed by dental pulp and liver sinusoidal ECs [105,129] and Ihh is expressed by ECs of the choroid in the mouse eye [130]. Finally, Hh ligands are shown to be expressed in tumor ECs [93,96,97].

Nevertheless, signaling pathways mediating Hh-regulation of intercellular junctions or VCAM-1 expression are unknown. For instance, the Hh canonical signaling involvement (i.e. Gli dependant transcription) remains controversial while several study have reported Gli1 overexpression in ECs upon Shh treatment [15,33,108], few others suggest that the Hh canonical signaling is not functional in ECs and that, on the contrary, Hh ligands signal through noncanonical signaling notably via the activation of the RhoA /ROCK [12,131,132,133] or PI3K/Akt pathways [12,134]. Upregulation of Gli1 in ECs may depends on the EC territory; indeed, Gli1 is shown to be over expressed either in brain [15,108] or embryonic ECs [33]. 

The primary cilium is suggested to be necessary for BBB integrity since zebrafishes deficient for cilia biogenesis have increased risk of developmental intracranial hemorrhage which can be rescued either by activation of the Hh pathway (PKA dominant negative or Sufu KD) or by the endothelial-specific re-expression of intraflagellar transport genes [135]. However, neither adherens (Cdh5) nor tight junction proteins (ZO-1) are modulated in *ift81^hi409^ mutant* [135].

Finally, overexpression of Gli3, a negative regulator of Hh canonical signaling, in cultured ECs promotes Cxcl1, Cxcl2, Cxcl8, and TYMP expression. However, it is suggested to act independently from the Hh canonical pathway [65].

## 6. Blood Vessel Maturation

### 6.1. Role of Hh Signaling in Mural Cell Recruitment and Differentiation

**Figure 8:**
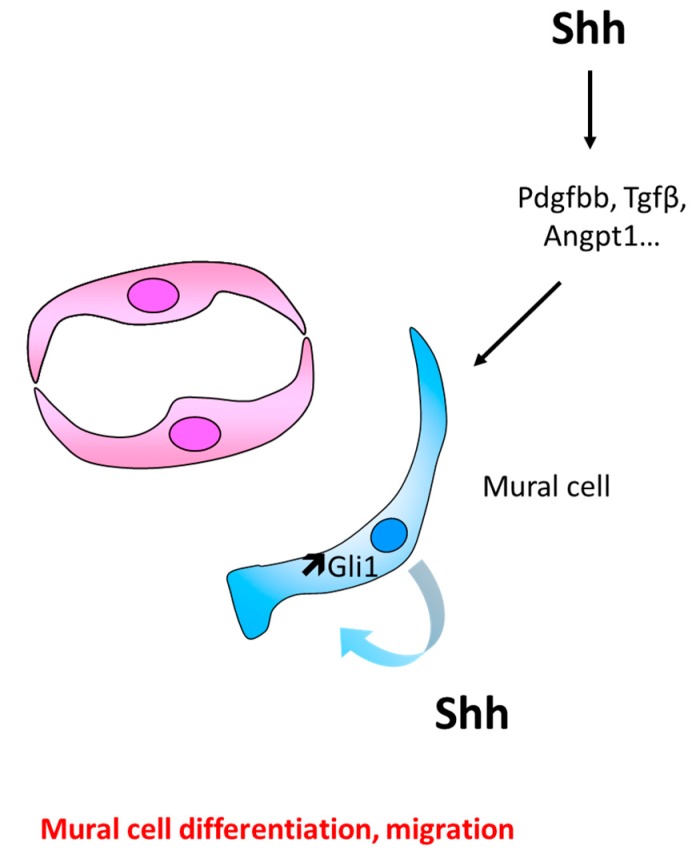
Schema representing the main cellular events involved in Hh-induced mural cell recruitment and differentiation. Shh may either promote mural cell differentiation and migration indirectly by upregulating Angpt1, Pdgfbb, or Tgfβ in unidentified cells or by upregulating Gli1 directly in ECs.

Shh was suggested to promote muscularization and maturation of blood vessels in 2004, in a study showing that Shh gene therapy promotes the formation of enlarged and more muscularized vessels in diabetic nerves [79]. Such observation has been confirmed in the ischemic heart in 2011 [136] and fibroblast growth factor 9 (FGF9) has been shown to increase Shh expression in SMCs and the formation of stable vessels [137]. However, Smo inhibition by Cyclopamine inhibits pericyte coverage of newly formed capillary in the mouse cornea [14] and in the retina of Akika diabetic mice, the decreased expression of Shh has been associated with the downregulation of Angpt1 expression, pericyte loss, and an increased capillary permeability [125] (Figure 8).

Interestingly, both igu mutant and cyclopamine-treated zebrafish embryos display decreased mesenchymal Angpt1 expression and hemorrhages located in multiple tissues throughout the body including the somites and pharyngeal area and the head. Tight junctions between ECs display normal morphology and size in mutant embryos. On the contrary, perivascular mural cells fail to make contact with ECs. These results suggest that hemorrhage in *igu^fo10a^* mutants is not due to an EC structural defect but to a pericyte defect [46]. Nevertheless, while Kolesova et al. reported that administration of 5E1 blocking antibodies in zebrafish embryos results in hemorrhages, distribution of SMCs in the vessel wall is unchanged [39].

Muscularization of new blood vessels is more likely the consequence of a direct response of mural cells (SMCs or pericytes) to Hh signaling stimulation, since both SMCs and pericytes express Shh, Ptch1, Smo and Gli1 [14,137,138,139]. Indeed, Shh is shown to promote SMC proliferation [140,141] and survival [138,139,141]. These effects are suggested to result from activation of Gli transcription factor directly [139,141,142], from modulation of Notch signaling [48,143,144] or activation of autophagy via Akt, phosphorylation [140]. Besides, Shh promotes SMC and pericyte migration [14,145,146] through ERK1/2 and PI3Kϒ activation [14]. Shh is also proposed either to maintain SMC differentiation [143] or to promote the differentiation of progenitor cells including Sca1+ adventitial resident stem cells [147,148,149] or bone marrow-derived mesenchymal stem cells [136] into SMCs. Similarly, in tooth, Shh activates Gli1 expression in peri-arterial cells, which give rise, at least in part, to NG2+ pericytes [150].

Differentiation of Sca1+ progenitor cells into SMCs has been shown to be mediated through Ptch1 and Gli2 [147]. Notably, one study reports that Shh, via Gli2 and Klf4, promotes VSMC dedifferentiation [151]. This last study is actually in accordance with studies reporting that Shh promotes SMC activation which is characterized by a proliferative and dedifferentiated state.

Finally, Shh increases the expression of factors promoting vessels stabilization such as Angpt1, platelet derived growth factor, BB dimer (Pdgf-BB), and transforming growth factor beta 1 (Tgfβ) [152,153].

#### Controversial Data

SIX homeobox 1 (Six) or EYA transcriptional coactivator and phosphatase 1 (Eya1) deficient embryos display an increased Shh expression especially because it fails to decrease after E16.5; these mutant embryos have severe vascular defects in the SMC compartment leading to the major vessel rupture and to hemorrhage. This is prevented when Shh expression is decreased (Eya1−/−; Shh+/− embryos) [154,155]. Moreover, embryos expressing a constitutively active form of Smo in the ovary Mullerian duct have increased CD31-labeled endothelial tube; nevertheless, these vessels are not covered by SMCs [57].

### 6.2. Role of Hh Signaling in Vascular Wall Remodeling

Besides participating in blood vessel maturation, Hh signaling, especially Shh and Scube2, has been shown to be overexpressed in injured arteries especially during intimal formation after carotid artery ligation [144,156,157] or autogenous vein grafts [142] and in the hypoxic lung [139]. In both conditions, Shh upregulation is associated with SMC activation and proliferation. Conversely, Shh, Ptch1, and SMC expression are decreased in aneurysmal tissue samples [143]. At the molecular level, Shh expression in SMCs is increased by hypoxia [139], growth factors such as Pdgf-BB [14,151], heart-type fatty acid-binding proteins [145] and C1q/TNF-related protein-5 [146]. Pdgf-BB-induced Shh expression is shown to depend on ERK1/2 signaling pathway [151]. On the contrary Shh expression in SMCs is downregulated by shear stress [48,141]. Finally, one study reports a decreased Hh signaling activity (i.e., Gli1, Gli2 and Hhip downregulation) after carotid artery ligation. This is associated with SMC dedifferentiation [158].

#### Discussion/Conclusion

Altogether, these data highlight the wide actions of the Hh signaling in vascular biology. First, the Hh signaling is essential for angioblast assembly into vascular tubes, i.e., vasculogenesis. This has been observed in the yolk sac [29] and during the formation of the aorta [38]. Hh signaling is also involved in angiogenesis in embryos and in adults especially under pathological conditions including cancer and ischemia [61] and participates in vascular maturation including arterial differentiation [16] and capillary muscularization. Finally, the Hh signaling has been shown to promote vascular integrity by maintaining endothelial intercellular junctions both at the BBB [15] and in peripheral tissues [110].

While cellular and molecular mechanisms underlying the vascular effects of the Hh signaling remain elusive, some consensuses appear. Both Hh-driven vasculogenesis and angiogenesis more likely involve activation of mesenchymal-type cells. Vasculogenesis depends on the Hh-induced BMP4 overexpression in these third cells while Hh-induced angiogenesis is mediated by Vegfa upregulation. On the contrary Hh-induced endothelial barrier tightness seems to depend on the Hh signaling activation in ECs themselves.

However some mechanisms remain to be elucidated. Indeed, differentiation of angioblast is also suggested to depend on EC-activation by Hh ligands directly [33] and Hh-induced arterial differentiation is either proposed to depend on Vegfa upregulation in a third cell [16] or to be a direct consequence of the Hh signaling activation in ECs [43]. So far, very few studies have used cell specific conditional KO mice to investigate Hh-regulation of blood vessel biology which limits the accuracy of mechanistical insights; studies using EC-specific Smo KO mice show that both vascularization of the embryonic lung [35] and ischemia-induced angiogenesis in the adult hindlimb does not involve activation of the Hh signaling in ECs [62,70], while Hh-induced endothelial tightness in the nervous system does [15,120]. Nevertheless, the possible involvement of type I Hh noncanonical signaling, which does not require Smo, has not been considered. While in vivo studies strongly support an indirect regulation of angiogenesis by Hh ligands, multiple in vitro studies have reported that treatment of ECs with Hh ligands promoted EC migration [12,33,119,132,133], proliferation [132], 2D capillary morphogenesis [12,38,118,119,131,132,159], and EC sprouting [118], altogether suggesting that Hh ligands may promote angiogenesis via a direct action on EC, at least in certain organs or conditions. 

Moreover, while Vegfa appears to be the main effector of Hh-induced angiogenesis, investigation of the regulation of Vegfa expression by the Hh signaling has been poorly investigated and limited to cancer cells. In breast cancer cells, Vegfa mRNA expression is shown to be specifically activated by a truncated form of Gli1 (splicing variant), while full-length Gli1 seems unable to bind Vegfa promoter and to activate its transcription [89]. Whether or not tGli1 is expressed in embryos or in adults in other cells than cancer cells is unknown.

Nevertheless, even if most studies agree in reporting that Hh signaling is proangiogenic, several conflicting data exist. Indeed, the endogenous Hh signaling has first been shown to promote ischemia-induced angiogenesis and systemic administration of Hh-blocking antibodies to decrease Vegfa mRNA levels and ischemic limb reperfusion [61]. However, later on, Gli3 knockdown which, on the contrary, induces increased activity of Hh canonical signaling, has also been shown to impair ischemic limb reperfusion [65,69]. Consistent with these two last studies, Shh deficiency has recently been associated to an indirect upregulation of Vegfa and to an accelerated ischemia-induced angiogenesis [66]. 

Therefore, further investigations are required to fully understand the role of the endogenous Hh signaling in the ischemic hindlimb. For instance, it would be interesting to compare the Hh signaling activity in mice administered with Hh-blocking antibodies and in mice deficient for Shh using reporter mice so that we could quantify the Hh signaling activity and Vegfa expression in different cell types. Indeed, while Pola et al. study involves the activation of the Hh signaling in fibroblast, studies conducted by Renault et al. investigated the role of the Hh signaling in myocytes [66,69]. It is important to note that Hh-blocking antibodies not only block Shh but also Ihh and Dhh activities [160]. 

Overall, the main reasons why mechanisms underlying the Hh action on the vasculature remain poorly understood are (1) lack of reliable, specific, and sensitive antibodies that could be used to detect expression of the Hh ligands and receptors so that we can identify Hh producing cells and Hh responding cells properly and (2) the limited amount of studies that have used tissue specific conditional KO mice. Moreover, the most widely used anti-Shh antibodies do also recognize Ihh and Dhh; this is the case of the rabbit anti-Shh antibodies (Santa-Cruz, sc-9024), goat anti-Shh antibodies (Santa-Cruz, sc-1194), and mouse anti-Shh antibodies (5E1, DHSB)). In fact, vascular effects induced by Hh ligands have been mainly attributed to Shh, while it may not be the case. For instance, Shh has been suggested to be the one promoting BBB tightness both in healthy and pathologic conditions [15], while a recent RNA single cell sequencing study shows that Dhh but not Shh and Ihh is expressed within the gliovascular unit in the healthy adult brain [161]. 

The proangiogenic potential of the Hh signaling agonists is of growing interest in the treatment of ischemic diseases especially myocardial infarction and peripheral artery diseases as pointed out in several reviews [17,18,19,20,21,22]. However, it is a prerequisite to fully understand mechanisms driving the exogenous Hh part in the setting of ischemia-induced tissue injury, (1) because the role of the endogenous Hh signaling is still unclear and discordant and (2) because of the potential carcinogenic effect of Shh. This is why it is necessary to design therapies targeting specific signaling molecules that would promote revascularization of ischemic tissue but not carcinogenesis.

Moreover, in the light of more recent studies, Hh signaling agonists may be beneficial in the setting of diseases in which endothelium integrity is compromised notably cardiovascular diseases like diabetic microangiopathies and cerebrovascular disorders, such as multiple sclerosis and HIV-induced encephalopathy.
